# *In Vivo* Expression of *Salmonella enterica* Serotype Typhi Genes in the Blood of Patients with Typhoid Fever in Bangladesh

**DOI:** 10.1371/journal.pntd.0001419

**Published:** 2011-12-13

**Authors:** Alaullah Sheikh, Richelle C. Charles, Nusrat Sharmeen, Sean M. Rollins, Jason B. Harris, Md. Saruar Bhuiyan, Mohammad Arifuzzaman, Farhana Khanam, Archana Bukka, Anuj Kalsy, Steffen Porwollik, Daniel T. Leung, W. Abdullah Brooks, Regina C. LaRocque, Elizabeth L. Hohmann, Alejandro Cravioto, Tanya Logvinenko, Stephen B. Calderwood, Michael McClelland, James E. Graham, Firdausi Qadri, Edward T. Ryan

**Affiliations:** 1 International Centre for Diarrhoeal Disease Research, Bangladesh (ICDDR,B), Dhaka, Bangladesh; 2 Division of Infectious Diseases, Massachusetts General Hospital, Boston, Massachusetts, United States of America; 3 Department of Medicine, Harvard Medical School, Boston, Massachusetts, United States of America; 4 Department of Microbiology and Immunology, University of Louisville School of Medicine, Louisville, Kentucky, United States of America; 5 The Vaccine Research Institute of San Diego, San Diego, California, United States of America; 6 Division of Biostatistics, Institute for Clinical Research and Health Policy Studies (ICRHPS), Tufts Medical Center, Boston, Massachusetts, United States of America; 7 Department of Microbiology and Molecular Genetics, Harvard Medical School, Boston, Massachusetts, United States of America; 8 Department of Immunology and Infectious Diseases, Harvard School of Public Health, Boston, Massachusetts, United States of America; Fondation Raoul Follereau, France

## Abstract

**Background:**

*Salmonella enterica* serotype Typhi is the cause of typhoid fever. It is a human-restricted pathogen, and few data exist on *S*. Typhi gene expression in humans.

**Methodology/Principal Findings:**

We applied an RNA capture and amplification technique, Selective Capture of Transcribed Sequences (SCOTS), and microarray hybridization to identify *S.* Typhi transcripts expressed in the blood of five humans infected with *S.* Typhi in Bangladesh. In total, we detected the expression of mRNAs for 2,046 *S.* Typhi genes (44% of the *S.* Typhi genome) in human blood; expression of 912 genes was detected in all 5 patients, and expression of 1,100 genes was detected in 4 or more patients. Identified transcripts were associated with the virulence-associated PhoP regulon, *Salmonella* pathogenicity islands, the use of alternative carbon and energy sources, synthesis and transport of iron, thiamine, and biotin, and resistance to antimicrobial peptides and oxidative stress. The most highly represented group were genes currently annotated as encoding proteins designated as hypothetical, unknown, or unclassified. Of the 2,046 detected transcripts, 1,320 (29% of the *S.* Typhi genome) had significantly different levels of detection in human blood compared to *in vitro* cultures; detection of 141 transcripts was significantly different in all 5 patients, and detection of 331 transcripts varied in at least 4 patients. These mRNAs encode proteins of unknown function, those involved in energy metabolism, transport and binding, cell envelope, cellular processes, and pathogenesis. We confirmed increased expression of a subset of identified mRNAs by quantitative-PCR.

**Conclusions/Significance:**

We report the first characterization of bacterial transcriptional profiles in the blood of patients with typhoid fever. *S.* Typhi is an important global pathogen whose restricted host range has greatly inhibited laboratory studies. Our results suggest that *S.* Typhi uses a largely uncharacterized genetic repertoire to survive within cells and utilize alternate energy sources during infection.

## Author Summary

*Salmonella enterica* serotype Typhi is the cause of typhoid fever and infects over 21 million cases and causes 200,000 deaths each year. *S.* Typhi only infects humans and this has greatly limited studies of *S.* Typhi pathogenesis. To study bacterial gene expression in human hosts, we used Selective Capture of Transcribed Sequences (SCOTS) and array hybridization to identify *S.* Typhi mRNAs expressed in the blood of 5 patients with *S.* Typhi infection. In total, we detected the expression of 2,046 *S.* Typhi genes (44% of the *S.* Typhi genome) in human blood; of these, 1,320 (29% of the *S.* Typhi genome) had significantly different levels of detection in human blood compared to *in vitro* cultures. Our results provide insight into *S.* Typhi pathogenesis, identifying both previously described and novel interactions occurring between host and microbe during the natural course of human infection. Further study of these genes, especially those of unknown function, may further our understanding of *S.* Typhi pathogenesis and aid in vaccine, diagnostic, and/or drug target development.

## Introduction

*Salmonella enterica* serotype Typhi is a Gram-negative bacterium and the cause of typhoid fever. Typhoid fever affects over 21 million people each year, killing 200,000 [1]. *S.* Typhi is a human-restricted pathogen and this has greatly limited studies of *S.* Typhi pathogenesis. Our current understanding of *S.* Typhi responses during infection is largely based on the study of murine models with the related bacterium *S.* Typhimurium (i.e., a bacteria that causes a typhoid-like illness in mice) [2], a separate mouse model of *S.* Typhi infection [3], and *ex vivo* macrophage and epithelial cell models of *S.* Typhi and *S.* Typhimurium [4] However, these studies have limitations, and do not fully replicate human disease. For instance, despite high sequence similarity, 13% of the genes in the *S.* Typhi genome are absent from *S.* Typhimurium, and the *S.* Typhi chromosome contains over 200 pseudogenes that *S.* Typhimurium does not [5], [6].

Here we report the application of an mRNA/cDNA capture and amplification technology, Selective Capture of Transcribed Sequences (SCOTS), combined with cDNA hybridization technology [7]–[12], to directly assess the gene expression profile of *S.* Typhi in the blood of humans with typhoid fever in Bangladesh. We previously applied this technology to *S.* Paratyphi A, the 2^nd^ leading cause of enteric fever, and detected expression of over 1700 bacterial genes during human infection [12]. Here we report the extension of this analysis to *S.* Typhi.

## Methods

### Ethics statement

This study was approved by the Ethical and Research Review Committees of the International Centre for Diarrhoeal Disease Research, Dhaka, Bangladesh (ICDDR,B) and the Human Research Committee of Massachusetts General Hospital; the study was conducted according to the principles expressed in the Declaration of Helsinki/Belmont Report. Written informed consent was obtained from all individuals or their guardians prior to study participation.

### Study subject selection and sample collection

Individuals presenting to the International Centre for Diarrhoeal Disease Research, Bangladesh (ICDDR,B) Hospital or the Kamalapur field site of ICDDR,B were eligible for enrollment if they met the following criteria at presentation: age of 1–59 years, fever duration of 3–7 days (≥39°C), no obvious focus of infection, and no alternate diagnosis. We collected 2 ml of venous blood from participants, immediately placed these specimens in TRIzol (Invitrogen Life Technologies, Carlsbad, CA) at a 1 (blood)∶2 (TRIzol) volume ratio, and stored the samples at −70°C for later analysis. We simultaneously obtained 3–5 ml of blood for microbiologic analysis using a BacT/Alert automated system. We sub-cultured positive bottles on MacConkey agar, and identified *S.* Typhi isolates using standard biochemical tests and reaction with *Salmonella*-specific antisera [13]. After we collected blood, we treated patients with parenteral ceftriaxone, oral ciprofloxacin, or oral cefixime for up to 14 days at the discretion of the attending physician.

### cDNA synthesis

To generate *S.* Typhi cDNA from blood samples, we used TRIzol-preserved blood of patients whose initial cultures were subsequently confirmed to grow *S.* Typhi. To create a corresponding *in vitro S.* Typhi cDNA sample for comparison, we grew each patient's bacterial isolate to mid-log growth phase (OD_600_ 0.45–0.6) in Luria Bertani (LB) broth, and preserved the samples in TRIzol at a 1 (mid-log culture)∶2 (TRIzol) volume ratio. We extracted total RNA from TRIzol preserved samples per the manufacturer's instructions (Invitrogen) and treated recovered RNA with DNase I on RNeasy columns (Qiagen Inc., Valencia, CA). We then converted 5 µg of total RNA into cDNA for each sample, as previously described with a few modifications [12]. Briefly, we used random priming (T-PCR) to obtain a representative amplifiable double-stranded cDNA population by using Superscript III (Invitrogen) with a conserved primer with a defined 5′ end terminal sequence and a random nonamer at the 3′ end [14]. We then synthesized second strands using the same primers and Klenow fragment (Invitrogen) according to the manufacturer's instructions, and then equilibrated samples based on 16S *S.* Typhi rRNA.

### Selective Capture of Transcribed Sequences (SCOTS)

We separated bacterial cDNA from host DNA using SCOTS, as previously described [12]. Briefly, we mixed denatured biotinylated *S.* Typhi gDNA with blocking ribosomal *S.* Typhi DNA, and added this denatured mixture to both *in vivo* and *in vitro* cDNA samples. After hybridizing samples overnight at 67°C, we captured biotinylated *S.* Typhi gDNA-cDNA hybrids using streptavidin-coated magnetic beads (Dynabeads M-280 streptavidin, Invitrogen), eluted captured cDNA with NaOH, PCR-amplified cDNA samples with conserved primers, and purified products using Qiagen PCR column purification kits. We performed three rounds of capture and amplification to separate *S.* Typhi cDNA from host DNA and to generate the cDNA mixture used for microarray hybridization.

### *Salmonella* microarray analysis

We labelled *in vivo* and *in vitro* cDNA recovered from SCOTS with Cy3 and Cy5, respectively, and hybridized these preparations to *Salmonella* ORF microarrays (version STv7S; McClelland Laboratory, Vaccine Research Institute of San Diego, CA, http://www.sdibr.org/Faculty/mcclelland/mcclelland-lab) in duplicate and with two dye reversals as previously described [12]. These microarrays contained gene-specific PCR-products of 4,600 ORFs from *Salmonella enterica* serotype Typhi CT18 (98.6% genome coverage) and 4,318 ORFs of strain Ty2 (98.0% genome coverage. The arrays also contained 1049 *S. enterica* ORFs absent from the *S.* Typhi genome. We used an equal amount of *in vivo* and *in vitro* Cy dye-labeled product on all slides for a given patient. We used ScanArray software (ScanArray express, version 3.0.1) to quantify signal intensities.

For each individual patient, we considered a gene to be detected *in vivo* if at least 2 of the 3 replicate gene spots on each of the four slides for that infected human was at least ten median absolute deviations greater than the median of spots on the microarray corresponding to genes absent from the *S.* Typhi CT18 or Ty2 genomes. For those genes we detected *in vivo*, we evaluated whether there was a difference in expression when compared to detection levels for *in vitro* grown organisms. For this latter statistical analysis, we included genes with a coefficient of variation in signal intensity less than 50% within an array, and employed repeated measures ANOVA (to within slide replicate spots) with type (*in vivo* versus *in vitro*) and dye effects to LOESS-normalized, log-transformed data. Those genes with a False Discovery Rate of less than 0.05 computed using Benjamini-Hochberg multiple testing adjustment and a 2-fold variation in signal intensity were considered differentially expressed *in vivo* versus *in vitro*. We deposited data in the NCBI Gene Expression Omnibus (GEO, www.ncbi.nlm.nih.gov/geo), accessible through GEO accession number GSE30565. We based functional classification of genes on J. Craig Venter Institute annotations (http://cmr.jcvi.org/tigr-scripts/CMR/CmrHomePage.cgi).

### Quantitative PCR analysis

We used quantitative real time PCR (RT-qPCR) to confirm microarray results for a subset of genes. We compared mRNA levels in the peripheral blood *(in vivo* sample) of infected patients (i.e. the 5 patients included in our SCOTS array analysis and 5 additional patients) to three *in vitro* culture replicates of a *S.* Typhi isolate (from Patient 1) grown to mid-logarithmic phase in LB (*in vitro* sample), as previously described [12]. To maximize the likelihood of detecting differences in gene expression in comparative samples, we selected eight representative genes from operons involved in intra-cellular invasion or survival (STY4609, *sopE*, invasion-associated secreted protein; STY3639, *trxA*, thioredoxin); alternate energy usage (STY2244, *pduB*, putative propranediol utilization protein; STY0417, *psiF*, phosphate starvation-inducible protein; STY2701, *eutN*, a putative ethanolamine utilization protein; STY0634, *fepC*, a ferric enterobactin transport ATP-binding protein); and bacterial adhesion (STY0207, *staA*, putative fimbrial protein and STY4543, *pilO*, putative pilus assembly protein), focusing on genes with high baseline signals and fold-increases by SCOTS-cDNA hybridization analysis comparing *in vivo* (high signal) to *in vitro* (low signal) samples. We also quantified by RT-qPCR the expression levels of two house-keeping genes that were predicted by SCOTS-cDNA hybridization to be equally expressed in *in vivo* and *in vitro* samples (STY0724, encoding a glutaminyl-tRNA synthetase, *glnS*; and STY3081, encoding an enolase, *eno*). We were unable to reproducibly assess expression levels of genes predicted by SCOTS-cDNA hybridization to be down-regulated in blood samples compared to *in vitro* grown organisms. To generate cDNA for quantitative RT-PCR from TRIzol-preserved samples, we used SuperScript II (Invitrogen) with random hexamers (Sigma, St. Louis, MO) according to the manufacturer's instructions, and performed RT-qPCR analysis using iQ SYBR Green Supermix reagent (Bio-Rad; Hercules, CA) and a CFX96 Real-time PCR detection system (Bio-Rad; Hercules, CA) as previously described [12]. Primers are listed in the Supplemental Table S1. We used no-template controls and samples lacking reverse transcriptase as baseline reactions for each sample. After calculating the threshold cycle (C_T_) in the low/linear portion of product curves, we quantified gene copy numbers using pGEM-T Easy-based plasmids (Promega, Madison, WI) containing the gene of interest. To calculate the control gene copy number, we used plasmid size and A260 readings, and normalized gene copy numbers based on cDNA copies of 16S rRNA. We assessed singularity of product species and size by melting curve analysis, as previously described [15].

## Results

### Patient samples

Of the 89 patients enrolled for blood sample collection, we identified 10 patients with confirmed *S.* Typhi bacteremia at the time of TRIzol-preserved blood collection. We performed SCOTS-cDNA hybridization screening analysis using samples from patients 1–5, and performed RT-qPCR on samples from patients 1–10, as sample quantity permitted.

### *S.* Typhi transcripts detected in the blood of infected humans

**Figure 1:**
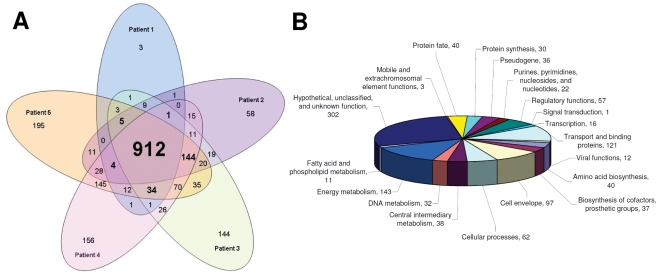
*S.* Typhi mRNA detected *in vivo* by SCOTS-microarray analysis. (A) Venn diagram of the 2046 *S.* Typhi transcripts detected *in vivo* by patient, and (B) functional classification of proteins encoded by the 1100 *S.* Typhi transcripts detected in 4 or more patients.

Using SCOTS-cDNA hybridization technology, we detected expression of 2046 *S.* Typhi genes in the blood of bacteremic patients. This represents approximately 44% of the *S.* Typhi ORFeome (Figure 1A, Supplemental Table S2). Of these, we detected expression of 912 genes in all 5 patients (45% of detected transcripts), and 1100 in at least 4 of the 5 patients (54% of detected transcripts).

The products encoded by the 1100 genes identified in 4 or more patients fell into a number of functional categories (Figure 1B). The most highly represented group were genes currently annotated to encode hypothetical proteins or proteins designated as unknown or unclassified. The next most highly represented groups were genes that encode products involved with energy metabolism, transport and binding, followed by genes encoding products of the cell envelope or associated with cellular processes and pathogenesis. Ninety-five of the 1100 genes were located within known *Salmonella* pathogenicity islands (SPI 1–7, 9, 10, 13, and 16), and 29 are known components of the PhoP regulon, a major virulence regulon in *Salmonella*, involved in intra-macrophage survival.

### *S.* Typhi transcripts with a different level of detection in *in vivo* versus *in vitro* bacterial samples

A total of 31 genes were detected in 4 or more patients *in vivo*, but not detected in any *in vitro* sample (Table 1). The majority of these genes are involved with survival in nutrient-limited conditions including *psiF*, a phosphate starvation-inducible protein; *bioF* and *thiG* involved in vitamin biosynthesis; *eutD*, *oadG*, and *pduB* involved in use of alternative carbon sources; and *fepD* involved in iron acquisition.

**Table 1:** *S.* Typhi transcripts detected only in *in vivo* samples.

CT18 Locus	Ty2 Locus	Gene	Function
**Biosynthesis of cofactors, prosthetic groups, and carriers**			
STY0828	t2092	*bioF*	8-amino-7-oxononanoate synthase
STY3725	t3471	*thiG*	thiamine biosynthesis protein
**Cell envelope**			
STY1609	t1379		hypothetical protein
STY4620	t4314	*nucD2*	putative lysozyme
**Central intermediary metabolism**			
STY0417	t2480	*psiF*	phosphate starvation-inducible protein PsiF
STY2702	t0393	*eutD*	putative phosphate acyltransferase
STY4773	t4468	*ppa*	inorganic pyrophosphatase
**Energy metabolism**			
STY1917	t1086	*hyaE*	hydrogenase-1 operon protein HyaE
STY2316	t0768	*manB*	Phosphomannomutase
**Fatty acid and phospholipid metabolism**			
STY2700	t0395	*eutE*	putative aldehyde dehydrogenase
**Protein synthesis**			
STY4360	t4067	*rplW*	50S ribosomal subunit protein L23
**Regulatory functions**			
STY3707	t3448	*yifE*	conserved hypothetical protein
**Transport and binding proteins**			
STY0065	t0058	*oadG*	oxaloacetate decarboxylase gamma chain
STY0123	t0110	*yabJ*	hypothetical ABC transporter
STY0636	t2276	*fepD*	ferric enterobactin transport protein FepD
STY2341	t0744	*mdtC*	putative RND-family transporter protein
**Hypothetical, Unclassified and Unknown proteins**			
	t3166		hypothetical protein
STY0321	t2569		Rhs-family protein
STY1058-60,1063-64,1069			putative prophage proteins
STY1323	t1640		conserved hypothetical protein
STY1548	t1434		conserved hypothetical protein
STY1732	t1256	*ydhZ*	conserved hypothetical protein
STY1916	t1087	*hyaD*	hydrogenase-1 operon protein HyaD
STY2244	t0835	*pduB*	putative propanediol utilization protein PduB
STY2608	t0487		conserved hypothetical protein
STY3448	t3185	*yraN*	conserved hypothetical protein

*S.* Typhi transcripts detected in the blood of 4 or more of 5 patients, but not in bacterial samples grown i*n vitro*.

**Figure 2:**
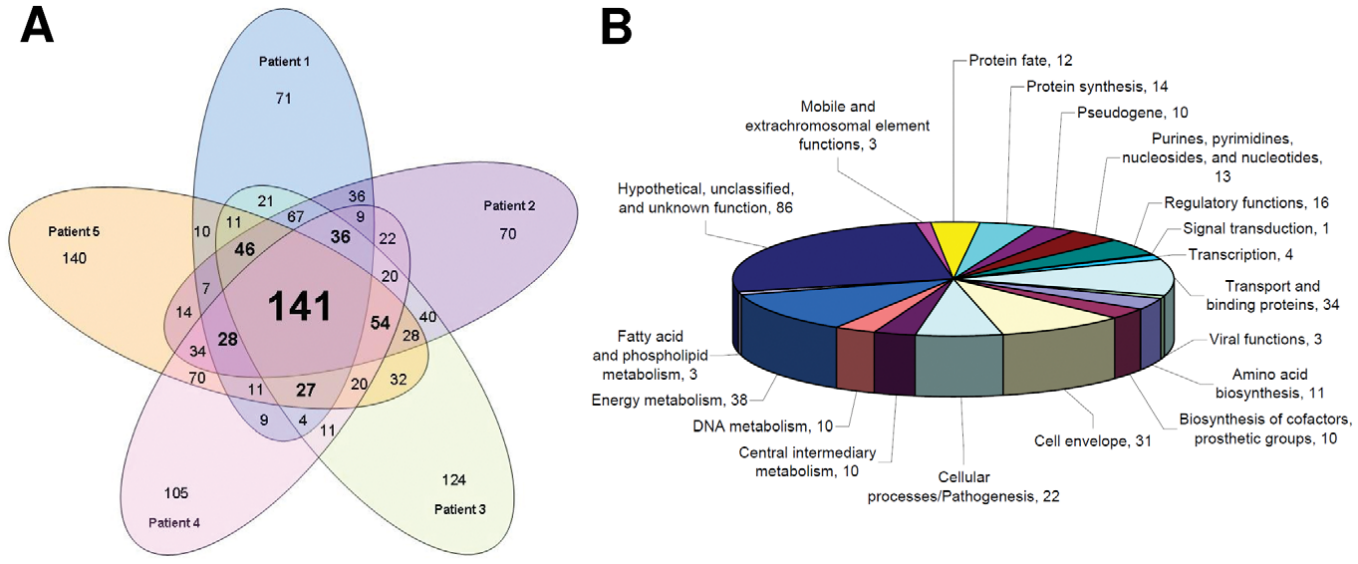
*S.* Typhi mRNA with different levels of detection *in vivo* versus *in vitro* bacterial samples by SCOTS-microarray analysis. (A) Venn diagram of the 1320 *S.* Typhi transcripts with significantly different levels of detection between *in vivo* and *in vitro* bacterial samples by patient, and (B) functional classification of proteins encoded by the 331 *S.* Typhi transcripts with significantly different levels of detection in 4 or more patients.

Of the 2046 transcripts detected in human blood samples, 1320 (representing 29% of S. Typhi ORFeome) had significantly different levels of detection in *in vivo* samples compared to bacterial samples grown *in vitro* (Figure 2A, Table S2). Detection levels for 141 transcripts were significantly different between *in vivo* and *in vitro* samples in all 5 patients, and 331 in at least 4 patients. These 331 encode products that fall into a number of functional categories (Figure 2B). The most highly represented group included proteins annotated as hypothetical, unknown, or unclassified. Other highly represented groups included energy metabolism, transport and binding, the cell envelope, and cellular processes and pathogenesis.

### Quantitative Real Time-PCR analysis

**Figure 3:**
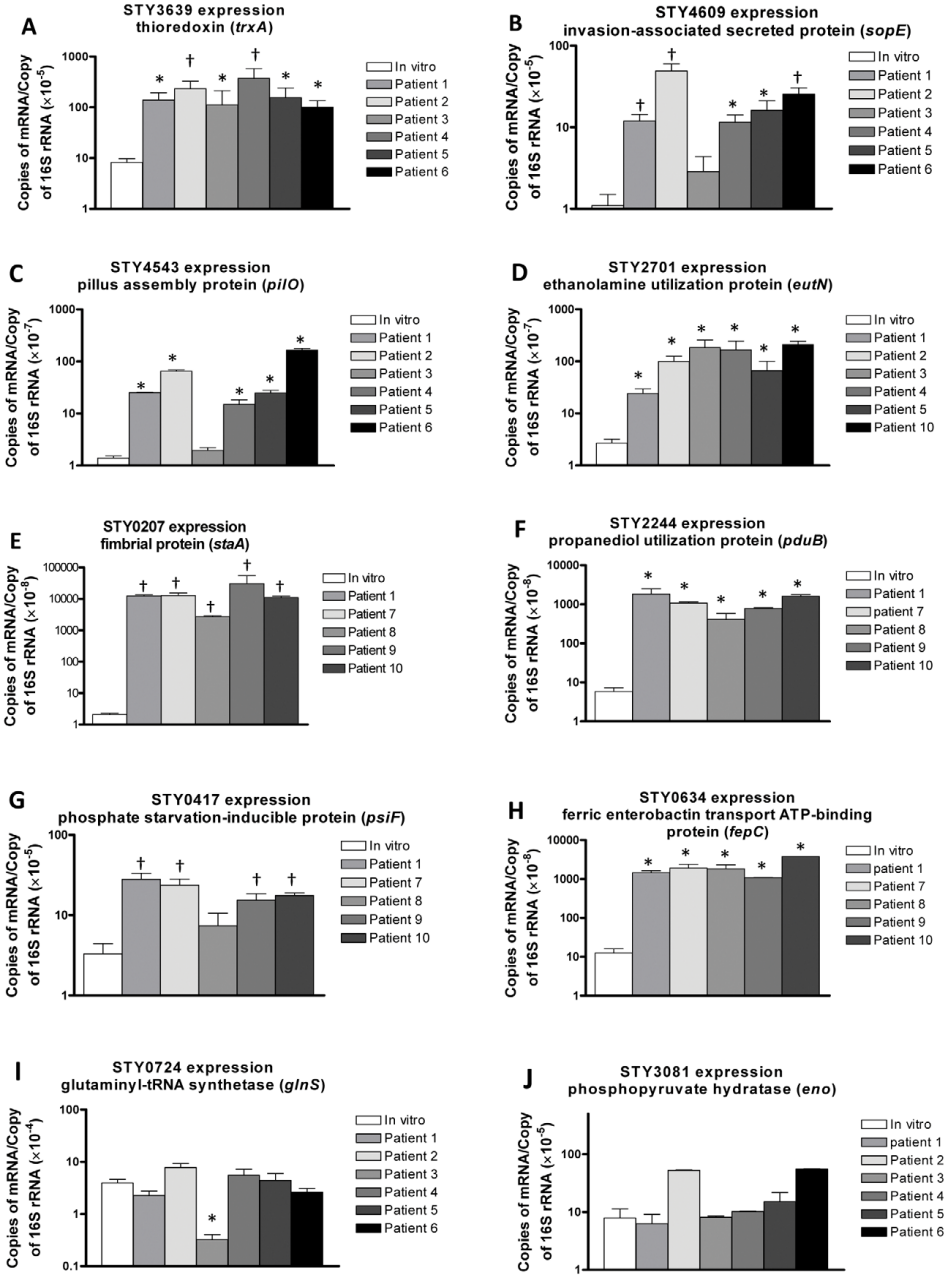
Quantitative RT-PCR *S.* Typhi mRNA expression profiles in human blood compared to *in vitro* bacterial samples. Quantitative real time-PCR analysis of *S.* Typhi genes (A–H) comparing RNA recovered from blood of bacteremic patients to an *in vitro* culture of the corresponding clinical isolate. Genes represented in A–H were identified by SCOTS-cDNA as being more highly expressed in human blood samples than in i*n vitro* grown organisms; genes I–J had equivalent levels of detection in i*n vivo* versus *in vitro* samples by SCOTS. Mean copies of mRNA per copy of 16S rRNA and standard error of the mean are presented. * p<0.05; †p<0.01. RT-qPCR was performed on a minimum of 5 patients as sample quantity permitted.

To confirm *S.* Typhi mRNA expression levels in human blood compared to *in vitro* grown bacteria, we used RT-qPCR to assess the copy number of the following eight genes that had high *in vivo* baseline reactivity as well as fold-change between *in vivo* and *in vitro* samples by SCOTS array analysis: thioredoxin, *trxA* (STY3639); a putative fimbrial protein, *staA* (STY0207); an invasion-associated secreted protein, *sopE* (STY4609); a putative propranediol utilization protein, *pduB* (STY2244); a putative pilus assembly protein, *pilO* (STY4543); an phosphate-inducible starvation protein, *psiF* (STY0417); a putative ethanolamine utilization protein, *eutN* (STY2701); and a ferric enterobactin transport ATP-binding protein, *fepC* (STY0634). Compared to expression levels in *in vitro* grown bacteria, we found increased expression of all 8 genes in the blood of infected humans, including in humans not analyzed by the SCOTS-cDNA hybridization screening protocol (Figure 3, A–H). As predicted by our SCOTS screening, we found no differences by RT-qPCR in the expression of housekeeping genes *glnS* (STY0724) and *eno* (STY3081) in blood versus *in vitro* bacterial samples (Figure 3, I-J).

## Discussion

*S.* Typhi is a human-restricted pathogen, the cause of typhoid fever, and a significant cause of global morbidity and mortality. Despite this, there are limited data on bacterial events within humans infected with *S.* Typhi. Here we describe the application of a cDNA capture-amplification approach combined with microarray hybridization technology to assess *S.* Typhi gene expression directly in the blood of infected humans. In total, we detected 2046 *S.* Typhi transcripts in human blood (45% of *S.* Typhi transcriptome); we detected 1100 in at least 4 of 5 patients. Two major virulence determinants of *Salmonella* are the ability to invade host cells and the ability to survive and replicate within host cells. The PhoPQ-two component regulatory system is involved in intra-macrophage survival and antimicrobial resistance [16], and *Salmonella* pathogenicity island-1 (SPI-1) and SPI-2 encode type three secretion systems (T3SSs) involved in invasion of host cells and intracellular survival and replication, respectively [17], [18]. In our analysis, we identified 29 genes involved in the PhoP regulon as more highly expressed in human samples, including the two component regulator itself, *phoPQ*; *virk*, a virulence protein; *mgtBC*, involved in magnesium transport; *pmrF*, a antimicrobial resistance protein; and *slyB*, an outer membrane lipoprotein [19], [20]. We also identified 95 genes located within previously described SPIs, including SPI-1 and 2, as well as genes within SPI-3–7, 9, 10, 13, and 16.

The role of SPI-1 in invasion of epithelial cells has been well established [21]. We detected a number of transcripts associated with SPI-1 genes, including a number that encode effector proteins injected into eukaryotic cells via the SPI-1 T3SS, such as SipB. We also detected a number of transcripts encoding SPI-1 T3SS effector proteins expressed from other SPIs, including *sopE* (expressed from SPI-7) and *sopB/sigD* (expressed from SPI-5); SopB/sigD is involved in creation and maintenance of the *Salmonella* Containing Vacuole (SCV), crucial to intra-cellular survival of *Salmonella* in eukaryotic cells [22]. Of note, we similarly identified SPI-1 transcripts in our recent analysis of *S.* Paratyphi A cDNA in the blood of infected humans in Bangladesh [12]. Our detection of these transcripts in the blood of infected humans builds upon recent suggestions that the SPI-1 T3SS is involved in pathogenic events beyond intestinal epithelial cell invasion during enteric fever [23]–[25]. In addition to *sopE*, we also detected transcripts from the Type IV pilus operon encoded within SPI-7, including *pilL*, *pilO*, *pilQ*, *pilR*, *pilU*, and *pilV*, which facilitates invasion of *Salmonella* into epithelial cells and monocytes [26], [27]. Identification of SPI-7 genes in our analysis is of particular interest since SPI-7 is absent from *S.* Typhimurium and *S.* Paratyphi A, but present in *S.* Typhi, *S.* Paratyphi C, and *S.* Dublin [28].

In addition to those associated with SPIs and the PhoPQ regulon, we detected transcripts from a number of virulence-associated *Salmonella* genes in human blood. These include aromatic amino acid biosynthesis pathway genes (*aroG*, *aroD*, *aroH*, *aroE*, *aroB*); mutations in this pathway have been the basis of live attenuated *S.* Typhi vaccines [29]. We also detected transcripts from genes involved in purine biosynthesis (*guaB*, *purG*, *purA*) [30] and divalent cation transport including Mg^2+^ (*corA*, *mgtBC*) [31]–[33], and Fe ^2+^ and Mn^2+^ uptake systems (*sitBC* and *mntH*) [34] that have all been associated with virulence in *Salmonella*.

In order to adapt to the intracellular environment, *Salmonella* must alter its metabolism to available nutrient and energy sources. We detected transcripts of genes involved in the use of alternative carbon sources, the coenzyme B12-dependent 1,2-propranediol utilization pathway (encoded by the *pdu* operon), and the ethanolamine utilization pathway (encoded by the *eut* operon). We also found these operons to be up-regulated in our analysis of *S.* Paratyphi A genes detected in the blood of humans [12], and mutations in these operons result in attenuation of virulence in *S.* Typhimurium infection models [35]–[37]. We also identified transcripts expressed from genes encoding three NiFe-uptake hydrogenases that have been associated with virulence in *S.* Typhimurium, including hydrogenase A, B and D [38]. Prior studies have shown that the *hya* and *hyd* operons are upregulated in murine and human phagocytes; *hya* genes are required for survival within macrophages, and both *hya* and *hyd* genes were detected in mice using the RIVET (Resolvase In-Vivo Expression Technology) reporter system that identifies genes expressed *in vivo* [39]. Our analysis shows that these genes are also expressed by *S.* Typhi during human infection. Other potential virulence-associated genes that we identified included genes involved in thiamine biosynthesis (e.g. *thiG*, *thiJ*, *abpA*), biotin biosynthesis (e.g. *bioB*, *bioF*, *kbl*), iron acquisition via siderophore biosynthesis (e.g. *iroA* gene cluster, *fes*, *fepECDB)*, and phosphate transport (*ugpBAEC* operon), many of which were also detected in our transcriptional analysis of *S.* Paratyphi A in infected humans [12].

In addition to survival in nutrient-limited conditions, *Salmonella* must also be able to survive the action of antimicrobial peptides, oxidative killing, and nitric oxide in various ecologic niches within the human body. We detected genes that may be involved in survival of stressful environments, including a number involved in antimicrobial resistance (e.g. *pqaB*, *virK*, *pmrF*, *smvA*, *bacA*, *emrA*, *mdtC*) [40]–[45], oxidative stress (e.g. *trxA*) [46], resistance to acid tolerance (e.g. *narZYWV* operon) [47], and genes involved in DNA recombination and repair (e.g. *recA*, *recBD*, *recN*, *recG*, *xthA*) [48]. Of note, the most highly represented group were genes currently annotated to encode hypothetical proteins or proteins designated as unknown or unclassified.

When comparing expression levels of *S.* Typhi genes detected in our analysis in humans to expression levels of *S.* Typhi genes in *in vitro* grown cultures, equilibrating for S. Typhi 16S rRNA, we noted differing levels of *S.* Typhi mRNA for 65% of the genes detected in humans. In total, 331 *S.* Typhi transcripts had significantly different levels of detection in at least 4 patients compared to *in vitro* cultures, and 141 had significant differences in all 5 patients compared to mRNA detected in *in vitro* cultures. Identified genes were involved in iron (*fepB*, *fepC*, *fepD*), thiamine (*thiG*), and biotin (*bioF*) metabolism; use of alternative carbon sources including ethanolamine (*eutB*, *eutC*, *eutD*, *eutA*, and *eutN*), oxacelatate (*oadAB* and *oadG*), and propranediol (*pduB* and *pduK*); and antimicrobial resistance (*bacA*, *mdtC*). We also identified these operons in our analysis of *S.* Paratyphi A, further supporting a potential role of these operons in the pathogenesis of enteric fever [12]. In addition, we identified 24 genes with significantly different levels of expression in *in vivo* compared to *in vitro* samples that are not present in the *S.* Typhimurium genome and may play an important role in *S.* Typhi pathogenesis, including genes encoded within the Type IV pilus cluster of SPI-7 (i.e. *pilO* and *pilL*), and fimbrial proteins *staA* and *steD*. Of note, the largest grouping of *S.* Typhi genes identified in our comparison encoded proteins of unknown or unclassified function.

Our findings are similar to prior *Salmonella* transcriptional analyses. We previously applied SCOTS-microarray analysis to *S.* Paratyphi A in the blood of infected humans, and the homologs of 75% of the bacterial transcripts identified in *S.* Paratyphi A infected patients were also identified in *S.* Typhi infected patients [12]. SCOTS analysis has also been previously applied to *S.* Typhi using an *ex vivo* macrophage model system by Faucher et al. [9]. Similar to our current analysis using blood of infected patients, the *ex vivo* analysis also detected transcripts of genes involved in intracellular survival including a number of genes encoded within SPI-2, *mgtBC* in SPI-3, the SPI-1 effector, *sopE*, and genes involved in antimicrobial peptide resistance. Both analyses suggested a role of SPI-1 beyond invasion of the intestinal epithelium and the potential role of alternative carbon sources in *S.* Typhi pathogenesis. In contrast to Faucher's analysis, we found higher levels of transcripts of genes involved in iron acquisition and transport *in vivo* including *fes*, *fhu*, *feo*, *iro*, and *ent*. Our detection of these genes may reflect a greater complexity or degree of iron-limitation in the blood of infected humans versus in a cultured macrophage model system.

To our knowledge there has not been a prior analysis of *S.* Typhi gene expression across the transcriptome in humans. Our results highlight potential survival adaptations of *S.* Typhi within the human host, including expression of genes required for utilization of alternative carbon and energy sources, divalent cation transport, antimicrobial resistance, and oxidative stress resistance, as well as many genes whose function is currently unknown. Further study of these genes, especially those of unknown function, may further our understanding of *S.* Typhi pathogenesis and aid in vaccine, diagnostic, and/or drug target development.

## Supporting Information

Table S1 qPCR Primer sequences.
(DOC)

Table S2 *S.* Typhi genes whose transcripts were detected in the blood of humans with typhoid fever.
(XLS)
