# Supplementary material for: In Vivo Expression of Salmonella enterica Serotype Typhi Genes in the Blood of Patients with Typhoid Fever in Bangladesh
Source: PLoS Negl Trop Dis. 2011 Dec 13;5(12):e1419. doi: 10.1371/journal.pntd.0001419 (PMC3236720; doi:10.1371/journal.pntd.0001419)
Supplement: Table S1 — qPCR Primer sequences. (DOC) [file pntd.0001419.s001.doc]

**Supplemental Table 1: qPCR Primer sequences**

| **qPCR primers** | Upstream | Downstream |
| --- | --- | --- |
| STY3639 (*trxA)* | GGATGAAATCGCTGACGAAT | TTGGCGTCGAGAAACTCTTT |
| STY4609 (*sopE*) | ATGACGTTTTTACGCCAAGC | GAGTCGGCATAGCACACTCA |
| STY4543 (*pilO*) | CCAGCAGTGTACACCCAATG | AGCAGGGTTGAAGGACAGAA |
| STY2701 (*eutN*) | GCAGATTCAAGGCCTACGAC | GCGGACGGTACATACGATTT |
| STY0207 (*staA*) | TTAGTGAGCGCAACAACCTG | CAGCAACGCCTTCAGTGATA |
| STY2244 (*pduB*) | CCACGGTTCGCTGATTATTT | CGGTGTACTGCAGCTCGATA |
| STY0417 (*ppa*) | GAAAATGCGTGAATGCAATG | CAGGCGCTCATAAACTTGCT |
| STY0634 (*fepC*) | TTTGATTGCATTACGCGAAG | ATACAGCGCAGCCCATAGAT |
| STY0724 (*glnS*) | TGATGAAGATCTGGCGAGTG | TTTTACCGGGTTGGTGTCAT |
| STY3081 (*eno*) | GCTCCGTCAGGTGCTTCTAC | GCGTCTTTGCCAAGAATAGC |
